# CD55 upregulation in astrocytes by statins as potential therapy for AQP4-IgG seropositive neuromyelitis optica

**DOI:** 10.1186/s12974-019-1448-x

**Published:** 2019-03-09

**Authors:** Lukmanee Tradtrantip, Tianjiao Duan, Michael R. Yeaman, Alan S. Verkman

**Affiliations:** 1 Departments of Medicine and Physiology, University of California, 1246 Health Sciences East Tower, 513 Parnassus Ave, San Francisco, CA 94143-0521 USA; 2 Department of Neurology, Second Xiangya Hospital of Central South University, Changsha, 410011 Hunan People’s Republic of China; 3 Department of Medicine, David Geffen School of Medicine, University of California, Los Angeles, CA 90024 USA; 4 Division of Molecular Medicine, Harbor-UCLA Medical Center, Torrance, CA 90502 USA

**Keywords:** NMO, Aquaporin-4, CD55, Astrocyte, Complement-dependent cytotoxicity, Statins

## Abstract

**Background:**

Neuromyelitis optica spectrum disorder (herein called NMO) is an inflammatory demyelinating disease that can be initiated by binding of immunoglobulin G autoantibodies (AQP4-IgG) to aquaporin-4 on astrocytes, causing complement-dependent cytotoxicity (CDC) and downstream inflammation. The increased NMO pathology in rodents deficient in complement regulator protein CD59 following passive transfer of AQP4-IgG has suggested the potential therapeutic utility of increasing the expression of complement regulator proteins.

**Methods:**

A cell-based ELISA was developed to screen for pharmacological upregulators of endogenous CD55 and CD59 in a human astrocyte cell line. A statin identified from the screen was characterized in cell culture models and rodents for its action on complement regulator protein expression and its efficacy in models of seropositive NMO.

**Results:**

Screening of ~ 11,500 approved and investigational drugs and nutraceuticals identified transcriptional upregulators of CD55 but not of CD59. Several statins, including atorvastatin, simvastatin, lovastatin, and fluvastatin, increased CD55 protein expression in astrocytes, including primary cultures, by three- to four-fold at 24 h, conferring significant protection against AQP4-IgG-induced CDC. Mechanistic studies revealed that CD55 upregulation involves inhibition of the geranylgeranyl transferase pathway rather than inhibition of cholesterol biosynthesis. Oral atorvastatin at 10–20 mg/kg/day for 3 days strongly increased CD55 immunofluorescence in mouse brain and spinal cord and reduced NMO pathology following intracerebral AQP4-IgG injection.

**Conclusion:**

Atorvastatin or other statins may thus have therapeutic benefit in AQP4-IgG seropositive NMO by increasing CD55 expression, in addition to their previously described anti-inflammatory and immunomodulatory actions.

## Introduction

Neuromyelitis optica spectrum disorder (herein called NMO) is an inflammatory demyelinating disease of the central nervous system. In seropositive NMO, immunoglobulin G autoantibodies against water channel aquaporin-4 (AQP4), called AQP4-IgG, bind to AQP4 on astrocytes and cause cytotoxicity largely by a complement-dependent mechanism [1–5]. Consequent inflammation, blood-brain barrier disruption, and oligodendrocyte injury result in demyelination and neurological deficit. Current therapy for NMO includes immunosuppressants, B cell depletion, and plasma exchange, and new therapeutics are in the development pipeline that target complement, cytokines, immune cells, and AQP4-IgG binding to astrocyte AQP4 [6–9].

Evidence for an important role of complement in seropositive NMO includes vasculocentric deposition of activated complement in NMO-affected human tissues [2, 10–12], complement-dependent NMO pathology in rodents following passive transfer of AQP4-IgG [13–15], and efficacy of the C5 complement inhibitor eculizumab in a preliminary clinical trial [16]. Additional evidence includes the increased NMO pathology in mice and rats lacking complement regulator protein CD59 following passive transfer of AQP4-IgG into the central nervous system [15, 17, 18]. NMO therapeutics that target components of the complement activation pathway can be associated with significant infectious and autoimmune side effects, as can agents that target B cells or other immune effectors [19, 20]. As an alternative approach, as motivated by the consequences of CD59 knockout [15, 17, 18], inhibition [15], and overexpression [21] in experimental animal models of NMO, here we investigate the therapeutic utility of pharmacological upregulation of complement regulator proteins in astrocytes.

We report a high-throughput screen to identify small molecule transcriptional upregulators of CD55 and CD59, the major complement regulator proteins in astrocytes. For screening, a human astrocyte-derived cell line was identified that endogenously expressed low to moderate levels of CD55 and CD59. Compounds emerging from the screen included statins, which were characterized for their mechanism of action in increasing CD55 expression in astrocytes and for their potential therapeutic utility in NMO.

## Materials and methods

### Materials

Purified recombinant AQP4-IgG (rAb-53, refs. [22, 23]) was provided by Dr. Jeffrey Bennett (Univ. Colorado, Denver). Human complement was purchased from Innovative Research (Novi, MI) and human (control) IgG from Pierce Biotechnology (Rockford, IL). GGTI-286 was purchased from Calbiochem (San Diego, CA), fasudil from Abcam (Cambridge, MA), and zaragozic acid A from Cayman Chemical Co. (Ann Arbor, MI). All other chemicals were purchased from Sigma-Aldrich (St. Louis, MO).

### Cell culture

U-251MG (NCI) and U-373MG (ATCC HTB-17) cells were cultured in Minimum Essential Medium (MEM) Eagle’s with Earle’s BSS supplemented with 10% fetal bovine serum, 2 mM glutamine, 1 mM sodium pyruvate, non-essential amino acid, 100 U/mL penicillin, and 100 μg/mL streptomycin at 37 °C in 5% CO_2_ 95% air. U-87MG (ATCC HTB-14) cells were cultured in the same medium but without sodium pyruvate and non-essential amino acids.

### Screening procedures

U-251MG cells were plated in black 96-well plates with a clear plastic bottom (Corning Inc., Corning, NY) at a density of 20,000 cells per well. Eighty wells contained test compounds, and the first and last columns of each plate were used for vehicle control (no test compound) and negative control (cells treated with 0.5 U/mL PI-PLC (Invitrogen, Carlsbad, CA)). After overnight growth to reach confluence, cells were washed twice with PBS, then 50 μL of culture medium was added to each well. Test compounds were added (0.5 μL of 2.5 mM DMSO stock) to each well to give 25 μM final concentration and incubated with cells at 37 °C overnight. After washing twice, cells were fixed in 4% paraformaldehyde for 15 min and then blocked with 1% BSA for 30 min. Then, 50 μL of a pre-mixed solution of anti-human CD55 antibody (NaM16-4D3, 1:200, Santa Cruz Biotechnology, Dallas, TX), anti-human CD59 antibody (YTH53, 1:200, Santa Cruz Biotechnology), Alexa Fluor-555 labeled goat anti-rat (A-21434, Invitrogen), and Alexa Fluor-647-labeled donkey anti-mouse IgG secondary antibody (A-31571, Invitrogen) (1:500 dilution) was then added and incubated with cells for 3 h. After washing four times with PBS containing 0.05% Tween-20, fluorescence was measured using a plate reader at excitation/emission wavelengths of 555/565 and 650/665 nm. Relative expression was computed as follows: (compound-negative control)/(DMSO-negative control).

### Astrocyte cell culture

Primary astrocyte cultures were generated from the brain cortex of neonatal rats at day 7 post-birth, as described [24] with modification. Briefly, the cerebral hemispheres were isolated and cortical tissue was minced and incubated for 15 min at 37 °C in 0.25% trypsin-EDTA. Dissociated cells were centrifuged and resuspended in Dulbecco’s modified Eagle’s medium (DMEM) containing 10% FBS and 1% penicillin/streptomycin, and grown at 37 °C in a 5% CO_2_ incubator. After confluence (8–10 days), flasks were shaken in a rotator at 180 rpm overnight to purify astrocytes and medium was replaced with DMEM containing 3% FBS and 0.25 mM dibutyryl cAMP. Cultures were maintained for an additional 2 weeks. Cultures contained > 95% astrocytes as seen by positive glial fibrillary acidic protein (GFAP) (AB5541, 1:1000, Millipore) immunofluorescence.

### Complement-dependent cytotoxicity (CDC)

Astrocyte cultures were trypsinized, plated onto 96-well plates at 20,000 cells/well, and grown for 48 h. Human complement and AQP4-IgG were added in Hank’s balanced salt solution (HBSS, pH 7.2; Invitrogen), and cells were incubated at 37 °C for 2 h for cytotoxicity measurement by the Alamar Blue assay (Invitrogen) as described [25]. For C3d immunostaining, astrocyte cultures were exposed to AQP4-IgG and C6-depleted serum for 2 h, then washed and fixed with 4% paraformaldehyde (PFA). C6-depleted serum was used to visualize complement activation without cell lysis from membrane attack complex formation. After blocking, cells were incubated with anti-human C3d (1:50, Santa Cruz Biotechnology), then washed and incubated with Alexa-Fluor 555-conjugated secondary antibody for 1 h (5 μg/mL, Invitrogen).

### Intracerebral injection model

Mice were housed and bred in the UCSF Laboratory Animal Resource Center. AQP4-IgG (7.5 μg) was delivered by intracerebral injection as described [13, 26] with modification. Adult wild-type male mice (age 8–10 weeks; weight 25–30 g) in a CD1 genetic background were anesthetized with ketamine (100 mg/kg) and xylazine (10 mg/kg) and mounted on a stereotaxic frame. A midline scalp incision was made, and a burr hole of 1-mm diameter was drilled on each side of the skull 0.5 mm anterior and 2 mm lateral to the bregma. A glass pipette with 40-μm diameter tip was inserted at a depth of 3 mm to infuse 7.5 μg AQP4-IgG (or control IgG) and 1 μL human complement in a total volume of 3 μL over 10 min by pressure injection. After injection, the glass pipette was kept in place for 10 min before slow withdrawal over 5 min to prevent leaking. At day 6, mice were deeply anesthetized and transcardially perfused with 200 mL heparinized PBS and 200 mL of 4% PFA in PBS. Brains were removed and post-fixed for 4 h in 4% PFA and cryoprotected in 20% sucrose. Serial frozen coronal sections (thickness 7 μm) were cut on a cryostat (Leica CM 1900, Leica Biosystem, Inc., Buffalo Grove, IL).

### Immunofluorescence

U-251MG and primary astrocyte cultures were grown on glass coverslips for 24 h, then incubated with test compound for another 24 h. Cells were then fixed in 4% PFA for 15 min. After blocking with 1% BSA in PBS, cells were incubated with primary antibody: anti-human CD55 (NaM16-4D3, 1:100), anti-human CD59 (YTH53, 1:700), anti-rat CD55 (RDIII-7 1:50, Hycult Biotech, Plymouth Meeting, PA), anti-rat CD59 (clone TH9, 1:50, LifeSpan Biosciences, Seattle, WA), or anti-human C3d (003-05, 1:50, Santa Cruz Biotechnology) for 1 h at room temperature. Cells were then washed with PBS and incubated with the appropriate species-specific Alexa Fluor-conjugated secondary antibody for 1 h (Alexa Fluor-488-labeled goat anti-rat (A-11006) and Alexa Fluor-555 labeled goat anti-mouse IgG secondary antibody (A-21424), 5 μg/mL each, Invitrogen). Slides were washed four times with PBS and mounted using Prolong™ Gold antifade reagent with DAPI (Invitrogen) counterstain for visualization of immunofluorescence on a Leica fluorescence microscope or Nikon confocal microscope.

Frozen sections of mouse brain, spinal cord, optic nerve, and skeletal muscle were post-fixed in 4% PFA for 5 min and incubated in blocking solution as described [18, 27]. Slides were then incubated for 2 h with antibodies against CD55 (NaM16-4D3, 1:50), CD59 (7A6, 2 μg/mL, Hycult Biotech), AQP4 (H-80, 1:200, Santa Cruz Biotechnology), C3d (003-05, 1:100, Santa Cruz Biotechnology), C5b-9 (aE11, 1:100, Santa Cruz Biotechnology), myelin basic protein (MBP, C-16, 1:100, Santa Cruz Biotechnology), ionized calcium-binding adaptor molecule 1 (Iba-1, 019-19741, 1:1000, Wako, Richmond, VA), or endothelial cell marker (isolectin B4-FITC conjugated, ALX-650-001F-MC05, 10 μg/mL, Enzo Life Sciences, Farmingdale, NY), followed by the appropriate secondary antibody for 1 h (Alexa Fluor-488 labeled donkey anti-rabbit (A-21206), Alexa Fluor-488-labeled donkey anti-goat (A-11055), Alexa Fluor-555-labeled donkey anti-mouse (A-31570), and Alexa Fluor-555-labeled donkey anti-rabbit IgG secondary antibody (A-31572), 5 μg/mL each, Invitrogen).

### Image analysis

Fluorescence signal was quantified using ImageJ (NIH). For measurements in cell cultures, mean pixel fluorescence intensity was measured using full-field images, with subtraction of background as determined using three or more areas devoid of cells. Relative fluorescence signal was reported as the ratio of drug-treated cells to DMSO-treated cells. Full-field images were also analyzed for measurements of CD55 immunofluorescence in tissues, in which background was determined after thresholding to exclude ~ 25% of the brightest pixels; robustness of the analysis was verified in an independent analysis that used thresholding to identify regions of interest around cell structures (not reported). For mouse brain, quantification of lesion area was done by image analysis as described [17] in which AQP4 and MBP immunonegative areas, and C5b-9 immunopositive areas, were defined by hand and areas quantified using ImageJ.

### RT-PCR

cDNA from reverse-transcribed mRNA isolated from U-251MG cells was PCR amplified with the following primers: 5′-TGACTGTGGCCTTCCCCCAGAT-3′ and 5′-GTGTTACATGAGAAGGAGATGG-3′, 168–640 (CD55), and 5′-TGACGGGGTCACCCACACTGTGCCCATCTA-3′ and 5′-CTAGAAGCATTGCGGTGGACGATGGAG GG-3′, 478–1128 (β-actin) [28] in an Applied Biosystems 2720 Thermal Cycler (Applied Biosystems, Foster City, CA). PCR conditions were at 94 °C for 5 min followed by 30 cycles of 94 °C for 30 s and 54 °C for 1 min, with final extension at 72 °C for 7 min. After amplification, the PCR products were run on a 2% agarose gel with ethidium bromide and photographed by Axygen Gel® Documentation System: Model GDBL-1000 (Fisher Scientific, Waltham, MA). The images were quantified using ImageJ; relative mRNA expression was calculated by the ratio of target gene expression to reference gene (β-actin).

### Statistics

Data are presented as mean ± SEM. Statistical analysis was performed using Prism 5 GraphPad Software package (San Diego, CA). Normal distribution of all data was confirmed by the Shapiro-Wilk test. Statistical comparisons were made using unpaired Student’s *t* test for comparisons between two groups. Differences were considered significant at *P* < 0.05 (* = *P* ≤ 0.05, ** = *P* ≤ 0.01).

## Results

### Complement regulator screen

**Fig. 1:**
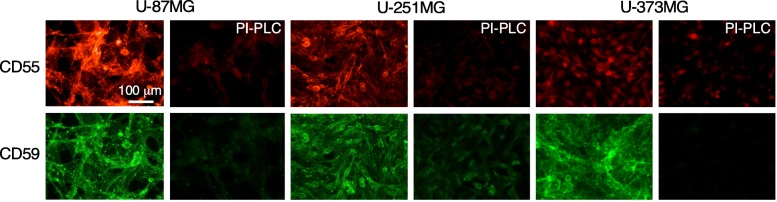
Complement regulator protein expression in human astrocyte cell lines. CD55 and CD59 immunofluorescence in U-87MG, U-251MG, and U-373MG cells. As control, cells were treated with PI-PLC to cleave the extracellular domains of CD55 and CD59. Representative of two sets of experiments

An assay suitable for high-throughput screening was established to identify compounds that upon many hours of incubation could increase the expression of CD55 or CD59 in astrocytes. Because compounds were sought that acted by a transcriptional mechanism, a human astrocyte cell line was chosen with low or moderate endogenous expression of CD55 and CD59 in order to produce measurable signals in the screening assay and in which baseline expression is sufficiently low to permit upregulation. The immortalized human astrocyte cell lines U-87MG, U-251MG, and U-373MG were tested. CD55 and CD59 immunofluorescence showed different levels of expression in the three cell lines (Fig. 1). Little fluorescence was seen in control cells following treatment with PI-PLC, which cleaves the extracellular antigen-containing portions of CD55 and CD59. U-373MG cells were not used for screening because of their very low CD55 immunofluorescence. U-87MG cells were not used for screening because they detached easily during washing steps and because of their high baseline CD55 expression that is likely to be near or at the maximum level. U-251MG cells were selected for screening based on their moderate expression of CD55 and CD59, which provided a measurable signal with sufficient room for upregulation.

**Fig. 2:**
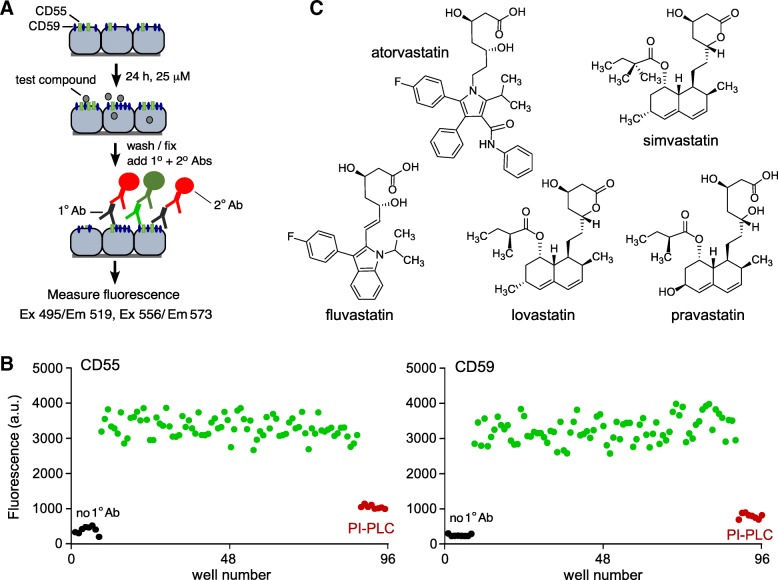
Screen for compounds that increase cell surface expression complement regulator proteins. **a** Schematic of 2-color cell-based ELISA for detection of cell surface CD55 and CD59 expression on U-251MG cells. **b** Examples of primary screen data as measured in 96-well plate format for immunodetection of CD55 (left) and CD59 (right). **c** Chemical structures of statins tested for CD55 upregulation

Figure 2a diagrams the cell-based screening assay in which suitable antibodies were chosen for two-color detection of CD55 and CD59 expression in the same cells without cross-talk. The assay used a mouse anti-human primary CD55 antibody and rat anti-human primary CD59 antibody, with appropriate fluorescent secondary antibodies. Examples of assay data are shown in Fig. 2b. PI-PLC strongly reduced fluorescence for both CD55 and CD59 detection, demonstrating assay specificity; *Z*-factor analysis showed assay reliability (Z’-factors 0.52 and 0.48, respectively). The assay did not include “positive controls” because there are no known compounds that upregulate CD55 or CD59 expression in astrocytes.

Screening was done on collections of approved and investigational drugs, and nutraceuticals, recognizing the benefits of drug repurposing for human clinical testing. The screen, which was done at a drug/nutraceutical concentration of 25 μM, did not produce compounds that significantly increased CD59 expression (cut-off 1.5-fold); however, compounds that increased CD55 expression by > 2-fold were identified, including several statins (atorvastatin, simvastatin, lovastatin, mevastatin) and phorbal 12-myristate 13-acetate. Because of their generally good brain penetration and established safety profile, further studies were done on statins. Figure 2c shows chemical structures of FDA-approved statins that were investigated, each of which contains a dihydroxyheptanoic acid component that is structurally similar to the 3-hydroxy-3-methyl-glutaryl-coenzyme A (HMG CoA) substrate in the cholesterol biosynthetic pathway.

### CD55 upregulation by statins

**Fig. 3:**
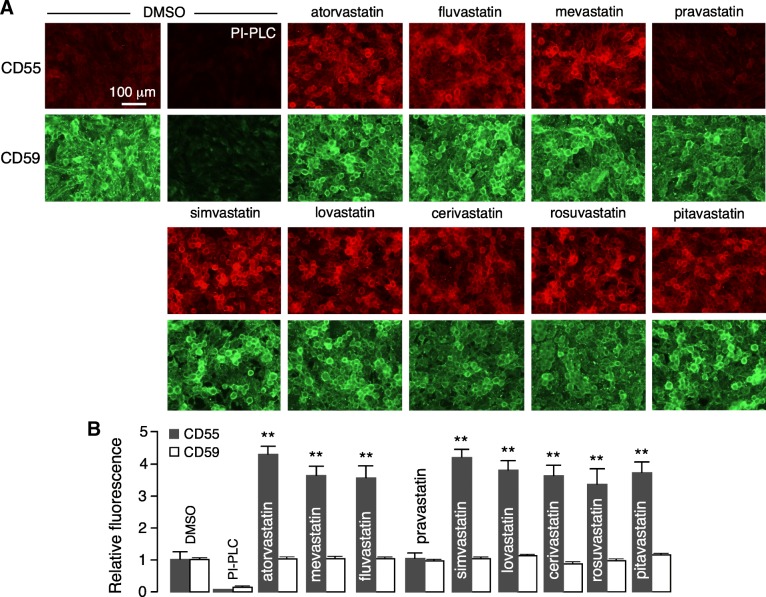
Statins upregulate astrocyte CD55. **a** CD55 and CD59 immunofluorescence in U-251MG cells after 24-h treatment with statins at 20 μM. PI-PLC control provided to show background fluorescence. **b** Summary of relative fluorescence as a measure of CD55 and CD59 cell surface expression, normalized to unity in the absence of statins (mean ± SEM, *n* = 7, **P < 0.01, unpaired Student’s *t* test, compared with DMSO vehicle)

U-251MG cells were incubated with each of the statins at 25 μM for 24 h. Figure 3a shows representative CD55 and CD59 immunofluorescence, with the fold-increased expression summarized in Fig. 3b. Most statins substantially increased CD55 expression, though none of the compounds increased CD59 expression. The broad activity of statins on CD55 expression suggests action on their well-known target, HMG-CoA reductase, rather than off-target effects. The lack of effect of pravastatin may be related to its high hydrophilicity and consequent low cell permeability. Atorvastatin, which is the seventh most commonly prescribed drug in the US, was selected for further studies because of its penetration into the central nervous system [29, 30], as well as its favorable immunosuppressive and immunomodulatory actions [31, 32], which are of potential therapeutic benefit in NMO.

**Fig. 4:**
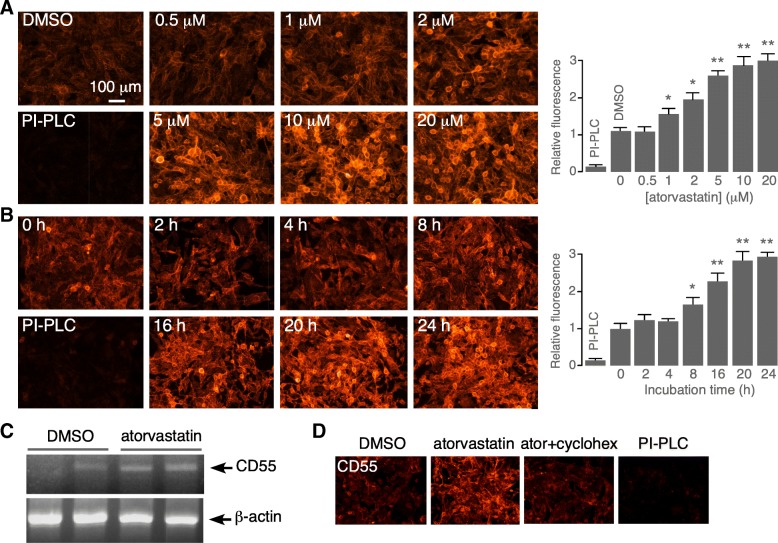
Concentration-dependence and kinetics of atorvastatin upregulation of astrocyte CD55 expression. **a** Atorvastatin concentration-dependence in U-251MG cells following 24-h incubation. Representative immunofluorescence shown at the left and summary at the right (mean ± SEM, *n* = 7, *P < 0.05, **P < 0.01, unpaired Student’s *t* test, compared with DMSO). **b** Time course of CD55 upregulation following incubation with 5 μM atorvastatin. Representative immunofluorescence shown at the left and summary at the right (mean ± SEM, *n* = 7, *P < 0.05, **P < 0.01, unpaired Student’s *t* test, compared with 0 h). **c** RT-PCR of CD55 and β-actin transcript expression following 48-h incubation of U-251MG cells with 1 μM atorvastatin. **d** CD55 immunofluorescence in U-251MG cell following 24-h incubation with 1 μM atorvastatin in the absence or presence of 3 μg/mL cycloheximide. Representative of two sets of experiments

Atorvastatin concentration-dependence data for CD55 upregulation in U-251MG cell cultures showed an EC_50_ of 1–2 μM at 24-h incubation time (Fig. 4a). A time course study at 5 μM atorvastatin showed slow onset of action, with a 50% increase in CD55 expression at 8–16 h, which is consistent with a transcriptional upregulation mechanism (Fig. 4b). In support of a transcriptional mechanism, RT-PCR showed significant increased CD55 transcript in U-251MG cells treated for 48 h with 1 μM atorvastatin, with comparable β-actin transcript (Fig. 4c) (fold increase comparing with vs. without atorvastatin, 1.6 ± 0.1, SEM, *n* = 3, *P* < 0.01). Cycloheximide block of protein synthesis largely prevented the increased CD55 expression by atorvastatin (Fig. 4d).

### Mechanism of CD55 upregulation by atorvastatin

**Fig. 5:**
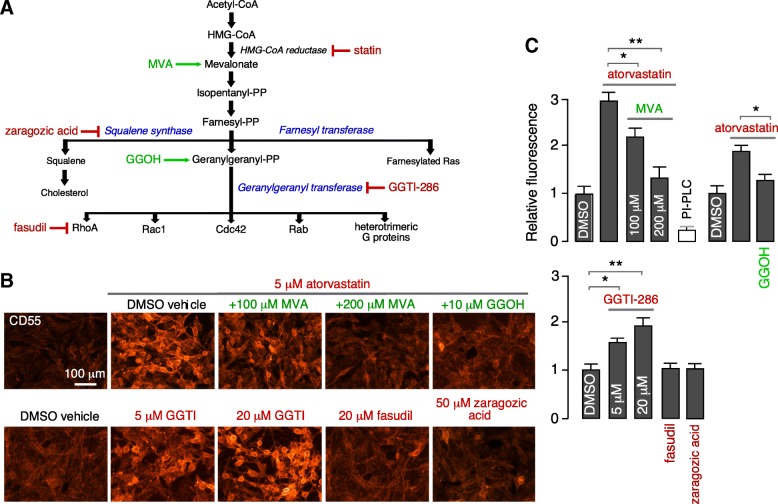
Mechanism of CD55 upregulation by atorvastatin. **a** Schematic of cholesterol biosynthesis pathway and sites of action of indicated agonists and inhibitors. Inhibitors color coded in red and activators in green. **b** CD55 immunofluorescence in U-251MG cells following 24-h incubation with indicated compounds. **c** Summary of relative fluorescence (mean ± SEM, *n* = 7, *P < 0.05, **P < 0.01, unpaired Student’s *t* test, comparison group as indicated)

Statins inhibit cholesterol biosynthesis as well as the formation of isoprenoid intermediates farnesyl pyrophosphate (farnesyl-PP) and geranylgeranyl pyrophosphate (geranylgeranyl-PP), which affects posttranslational modification of a variety of signaling proteins, including small GTPase RhoA, Cdc42, Rac1, Rab, and heterotrimeric G proteins (Fig. 5a). Selective modulators of components of the acetyl-CoA signaling pathway were tested for their effects on CD55 expression. As shown in Fig. 5b and c, inclusion of mevalonic acid (MVA) with atorvastatin for 24 h prevented the atorvastatin effect, supporting the conclusion that atorvastatin upregulation of CD55 involves inhibition of HMG-CoA reductase, as mevalonate bypasses the enzyme block. The squalene synthase inhibitor zaragozic acid did not increase CD55 expression, indicating that the atorvastatin effect is not related to its cholesterol-lowering action. Geranylgeraniol (GGOH), an alcohol precursor of geranylgeranyl pyrophosphate (geranylgeranyl-PP), largely blocked the atorvastatin-induced CD55 upregulation, suggesting that action of atorvastatin on the geranylgeranyl transferase pathway is responsible for CD55 upregulation. In support of this conclusion, the geranylgeranyl transferase inhibitor GGTI-286 (in the absence of atorvastatin) recapitulated the CD55 upregulation seen with atorvastatin. The RhoA inhibitor fasudil did not increase CD55 expression.

### Atorvastatin increases CD55 expression in astrocyte cultures and mouse brain

**Fig. 6:**
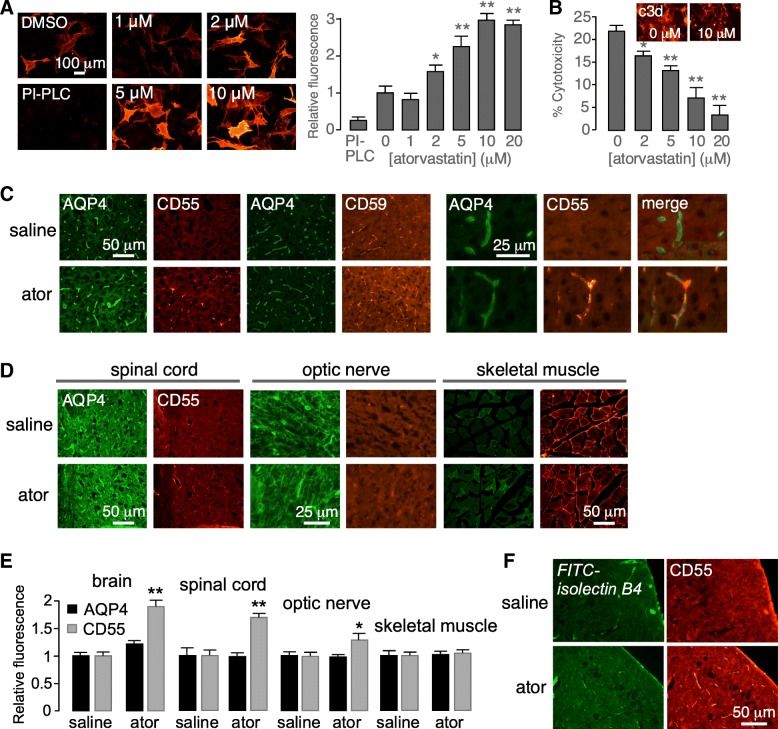
Atorvastatin increases CD55 expression in primary astrocyte cultures and murine brain. **a** Astrocyte cultures were incubated with atorvastatin for 24 h. Representative immunofluorescence shown in the left panel and summary data in the right panel (mean ± SEM, *n* = 6, *P < 0.05, **P < 0.01, unpaired Student’s *t* test, compared with DMSO (0 μM)). **b** Complement-dependent cytotoxicity in murine astrocyte cultures exposed to 5% human complement and 20 μg/mL AQP4-IgG following 24-h incubation with atorvastatin (or vehicle control) (mean ± SEM, *n* = 4, *P < 0.05, **P < 0.01, unpaired Student’s *t* test, compared with DMSO (0 μM)). Inset indicates C3d immunofluorescence in cells exposed to AQP4-IgG and c6-depleted complement. **c** (left) CD55 or CD59 (red) and AQP4 (green) immunofluorescence in cerebral cortex of control and atorvastatin-treated (10 mg/kg daily for 3 days) mice. (right) Higher magnification micrographs showing colocalization of AQP4 and CD55. **d** CD55 and AQP4 immunofluorescence of the spinal cord, optic nerve, and skeletal muscle from control and atorvastatin-treated mice. Representative of four mice per group. **e** Summary of relative fluorescence as a measure of AQP4 and CD55 cell surface expression, normalized to unity in the absence of atorvastatin (mean ± SEM, *n* = 6, **P < 0.01, unpaired Student’s *t* test, compared with saline). **f** FITC-Isolectin B4 (green) and CD55 (red) immunofluorescence in cerebral cortex of control and atorvastatin-treated mice. Representative of four mice per group

Atorvastatin upregulation of CD55 was investigated in primary astrocyte cultures and in mice in vivo. Primary astrocyte cultures were generated from neonatal rat brains and differentiated by inclusion of dibutyryl cAMP in the culture medium. Atorvastatin for 24 h increased CD55 expression by up to ~ 3-fold, without significant effect on CD59 expression (Fig. 6a). CD55 immunofluorescence showed a somewhat heterogeneous response from cell to cell, both in CD55 expression at baseline and after atorvastatin treatment. To determine whether CD55 upregulation in rat astrocyte cultures was protective against complement-dependent cytotoxicity, cytotoxicity was measured in control cells and cells incubated for 24 h with atorvastatin prior to addition of AQP4-IgG and human complement. Atorvastatin significantly reduced cytotoxicity in a concentration-dependent manner (Fig. 6b). C3d immunofluorescence of atorvastatin-treated astrocyte cultures exposed to AQP4-IgG and C6-depleted complement was greatly reduced (Fig. 6b, inset), consistent with the cytoprotective action of atorvastatin.

For experiments in vivo, mice were orally administered atorvastatin at 10 mg/kg daily for 3 days, a dose regimen similar to that used in prior in vivo brain studies in mice [33–35]. CD55 immunofluorescence showed increased CD55 immunofluorescence in the brain, optic nerve, and spinal cord of atorvastatin-treated mice, though no significant effect in skeletal muscle (Fig. 6c–e). CD59 expression was similar in control and atorvastatin-treated mouse brain (Fig. 6c, left). The pattern of CD55 expression in atorvastatin-treated mouse brain was similar to that of AQP4, the astrocyte target of NMO autoantibody (Fig. 6c, right) and that of an endothelia cell marker (FITC-Isolectin B4) (Fig. 6f); however, by light microscopy the relative increase in CD55 expression in astrocytes versus endothelia and other brain cells cannot be resolved. The expression of AQP4 in central nervous system tissues and skeletal muscle was similar in the control and atorvastatin-treated mice (Fig. 6c–e).

### Atorvastatin reduces pathology in an experimental mouse model of NMO

**Fig. 7:**
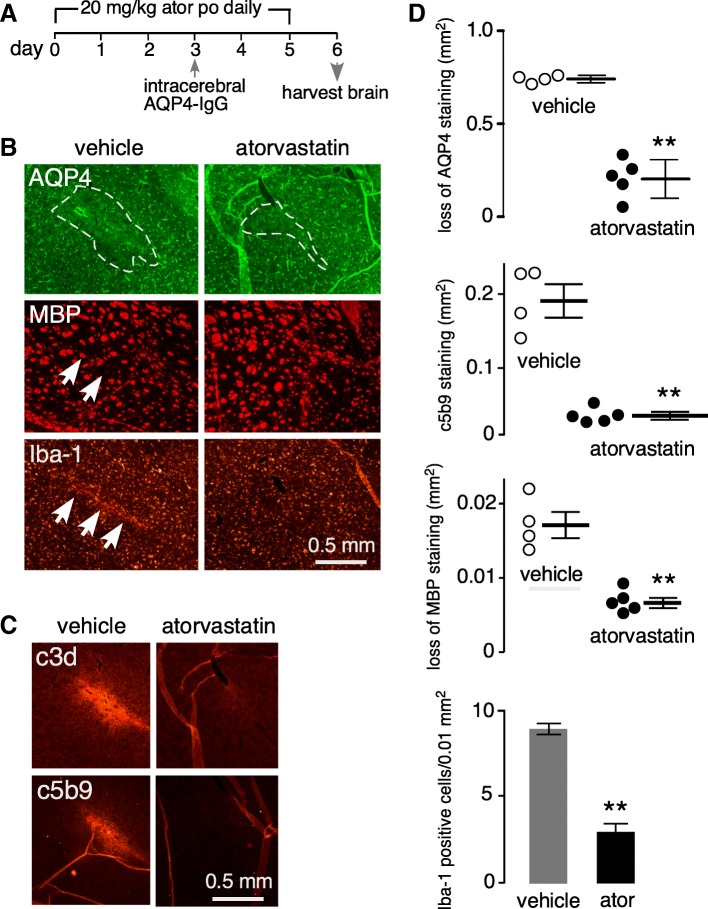
Atorvastatin reduces NMO pathology in an experimental animal model of NMO produced by passive transfer of AQP4-IgG. **a** Experimental protocol. Mice received atorvastatin 20 mg/kg/day for 3 days, or saline vehicle, prior to intracerebral injection of 7.5 μg AQP4-IgG and 1 μL human complement. Mice were sacrificed on day 6. **b** Immunofluorescence of AQP4, MBP, and Iba-1 in the brain at day 6 showing AQP4-IgG-injected hemisphere from control and atorvastatin-treated mice. Representative of experiments on 4–5 mice per group. **c** Immunofluorescence of activated complement proteins C3d and C5b-9 at day 6 from control and atorvastatin-treated mice. Representative of staining done on 4–5 mice per group. **d** Summary of lesion areas (AQP4, c5b-9, MBP, in mm^2^) and Iba-1 cell positivity (positive cells per 0.01 mm^2^), showing data from individual mice (mean ± SEM, *n* = 4–5, **P < 0.01, unpaired Student’s *t* test, compared with vehicle)

Atorvastatin efficacy in reducing NMO pathology was tested in an established experimental animal model of NMO. As diagrammed in Fig. 7a, mice received atorvastatin at 20 mg/kg daily for 3 days prior to intracerebral injection of AQP4-IgG and human complement, with control mice receiving saline instead of atorvastatin [13, 26, 36]. Atorvastatin was continued daily until sacrifice on day 6 at which time brains were processed for immunofluorescence. Figure 7b shows reduced loss of AQP4 and MBP, and reduced Iba-1 immunofluorescence, in the brain of atorvastatin-treated mice in a region surrounding the AQP4-IgG injection site compared with vehicle-treated mice. These findings demonstrate reduced astrocytopathy (AQP4), myelin loss (MBP), and inflammation (Iba-1) with atorvastatin treatment. Lesion size, as quantified by the areas of AQP4 and MBP loss, was significantly reduced in atorvastatin-treated mice (Fig. 7d). There was also an increase in immunofluorescence of markers of complement activation (C3d, C5b-9) (Fig. 7c, d) and inflammation (Iba-1) (Fig. 7d).

## Discussion

The purpose of this study was to identify a drug or nutraceutical that increases the expression of complement regulator protein(s) in astrocytes, for possible repurposing in NMO. The motivation for this work is the central importance of complement-mediated cytotoxicity in AQP4-IgG seropositive NMO, and the predicted importance, as demonstrated in experimental animal models [15, 17, 18] and by theoretical considerations [37], of complement regulator proteins in modulating complement action on target cells. Though there has been considerable research on complement mechanisms in NMO pathogenesis and on the development and testing of complement-targeted therapeutics [16, 25, 38], there has been relatively little work on complement regulator proteins in NMO. Increasing the expression or activity of complement regulator proteins on target cells is a logical extension of complement inhibitor therapy for NMO, with the potential advantage of avoiding the immunosuppressive actions of global complement inhibition and their concomitant side effects. We found evidence here for atorvastatin as a transcriptional upregulator of CD55 in astrocytes by a mechanism that involves the geranylgeranyl transferase pathway. The demonstrated consequences of CD55 upregulation included inhibition of complement-dependent cytotoxicity in astrocyte cultures in vitro and reduced pathology in an experimental mouse model of NMO in vivo.

Various physiological factors and drugs have been reported to increase expression of complement regulator proteins in human endothelial cells in the umbilical vein, skin, and aorta, which was proposed to have relevance to inflammatory diseases involving complement, including atherosclerosis and rheumatoid arthritis [39–43]. Physiological factors, including TNF-α, interferon-γ, and basic fibroblast growth factor (bFGF), were reported to increase CD55 expression by a protein kinase C (PKC)-independent pathway, whereas the vascular endothelial growth factor (VEGF) was reported as PKC-dependent [39, 40, 42]. Drugs including atorvastatin and simvastatin have been reported to increase complement regulator protein expression in endothelia (41, 43), which was proposed to explain the anti-inflammatory effect of statins on the cardiovascular system apart from their cholesterol-lowering action [44, 45].

Statins have pleotropic biological actions, several of which are of a potential benefit in NMO. Statin inhibition of HMG-CoA reductase blocks cholesterol biosynthesis, its well-described action as a lipid-lowering drug. Statins also inhibit biosynthesis of the isoprenoid intermediates farnesyl pyrophosphate and geranylgeranyl pyrophosphate [31, 44]. Statin-induced upregulation of CD55 in endothelial cells, and consequent protection against complement-mediated injury, was reported to involve inhibition of geranylgeranyl transferase (GGTase) and RhoA [41, 46]. The mechanistic studies here in Fig. 5b suggest that CD55 upregulation by atorvastatin in astrocytes also involves GGTase inhibition. Atorvastatin has also been reported to cause CD59 upregulation in hypoxia in a model of rheumatoid arthritis by a mechanism involving nitric oxide-dependent GGTase inhibition [43].

Several GGTase inhibitors (GGTIs) are in development as potential anticancer drugs [47–49]. GGTI-286, a potent and selective GGTase inhibitor, is in preclinical development, and GGTI-2418 is in a Phase I clinical trial for treatment of breast cancer and multiple myeloma [47–52]. Our data here support selective inhibition of GGTase as the mechanism of stain-induced CD55 upregulation in astrocytes. GGTIs may thus provide an alternative approach to increase CD55 expression without altering cholesterol biosynthesis. We note, however, that although a large body of literature supports the safety of statins, the safety of drugs that target other steps of the cholesterol biosynthesis pathway must be demonstrated. In addition, there are theoretical adverse effects of increasing complement regulator expression such as impairment of immune surveillance in cancer.

The ability of drugs to cross the blood-brain barrier is an important consideration in NMO therapy. The penetration of statins into the central nervous systems varies with their structure and lipophilicity. Lipophilic statins (atorvastatin, lovastatin, fluvastatin, pitavastatin, simvastatin) can penetrate the blood-brain barrier passively, while the hydrophilic statin pravastatin does not [29, 30]. Among the lipophilic statins, lovastatin has been reported in rat studies to best cross the blood-brain barrier [29]. However, lovastatin and simvastatin are inactive lactone prodrugs that require in vivo hydrolysis to give the pharmacologically active form. Atorvastatin is an active drug with high cellular and blood-brain barrier penetration. Atorvastatin has been reported to have neuroprotective effects in animal models of brain pathology associated with neuroinflammation, including traumatic brain injury and experimental autoimmune encephalomyelitis [33, 35]. Atorvastatin has also been reported to have immunomodulatory actions involving alteration of T and antigen-presenting cell (APC) function, and inhibition of immune cell invasion [31–33]. These immunomodulatory effects of atorvastatin may be beneficial in NMO, apart from its action on complement regulator expression.

An atorvastatin dose of 10 -20 mg/kg/day orally was chosen here for in vivo mouse experiments. This dose was based on pharmacokinetic data and prior studies of neuroprotection in rodents in which 10–50 mg/kg atorvastatin was needed to achieve similar plasma concentrations as in humans [30, 33–35, 53–55], in which the maximum recommended daily dose is 80 mg/day, or ~ 1–2 mg/kg. Atorvastatin may thus confer neuroprotection in humans at the typically used human doses.

Questions in targeting complement regulator proteins for NMO therapy include which complement regulator proteins to target and on what cell types and the magnitude of upregulation needed to confer clinical benefit. There are available data regarding CD59 in NMO from experimental animal models. Transgenic knockout of CD59 greatly increases the susceptibility of mice and rats to development of NMO pathology following passive transfer of AQP4-IgG into central nervous system tissues [15, 17, 18]. CD59 upregulation by lentivirus is associated with reduced NMO pathology [21]. Mathematical modeling showed that theoretical increases in CD59 expression could prevent cell swelling induced by AQP4-IgG and human complement [37]. Upregulation of CD55, but not of CD59, would inhibit the generation of complement anaphylotoxins (C3a, C4a, and C5a), as CD55 inhibits the assembly and accelerates the decay of C3- and C5-convertase. Additional potential advantages of upregulation of CD55 vs. CD59 for NMO therapy include the greater-fold increase in CD55 expression possible because of its low baseline expression compared to CD59 and inhibition of a different step in the complement pathway and hence possible synergy with CD59. Another theoretical advantage is the ostensible difference in the subcellular distribution of CD55 vs. CD59, as CD59 has been reported to be expressed at relatively lower levels in astrocyte end-feet where AQP4 is concentrated [56].

Though the data herein provide proof of concept for the potential therapeutic utility of CD55 upregulation by atorvastatin in seropositive NMO, several limitations are noted. The demonstration of CD55 upregulation by atorvastatin in astrocyte cultures and mouse brain does not ensure that clinically significant CD55 upregulation will occur in NMO-affected tissues in humans at tolerated doses of atorvastatin. As atorvastatin inhibits cholesterol biosynthesis, the possibility exists, at least theoretically, for inhibition of remyelination [57, 58], which might account for the inconclusive benefit of statin treatment in multiple sclerosis [59]. Finally, upregulation of CD55 alone does not target other effectors of NMO pathogenesis, such as AQP4-IgG production and levels, antibody-dependent cellular cytotoxicity, the peripheral complement system, and T cell responses, though atorvastatin could be combined with other currently used or investigational NMO therapeutics such as immunosuppressants, plasma exchange, complement inhibitors, and cell-targeted drugs.

## Conclusions

In conclusion, pharmacological upregulation of complement regulator proteins such as CD55 represents a novel approach for therapy of AQP4-IgG seropositive NMO. Since upregulation of complement regulator proteins does not interfere with the classical, alternative, or lectin complement pathways, the side-effect profile might be relatively minimal. Upregulation of CD55 expression by atorvastatin is predicted to not only inhibit formation of the complement membrane attack complex on astrocytes and nearby bystander cells, but also inhibit the formation of complement anaphylotoxins that promote inflammation and cellular injury. Clinical evaluation of atorvastatin in seropositive NMO therefore seems warranted based on its established safety profile and multiple potential beneficial actions. Retrospective analysis of NMO patients treated with statins for other indications may be informative as well.

## Abbreviations

AQP4: Aquaporin-4
AQP4-IgG: Aquaporin-4-immunoglobulin G
CDC: Complement-dependent cytotoxicity
DMEM: Dulbecco’s modified Eagle medium
farnesyl-PP: Farnesyl pyrophosphate
FBS: Fetal bovine serum
geranylgeranyl-PP: Geranylgeranyl pyrophosphate
GFAP: Glial fibrillary acidic protein
GGTase: Geranylgeranyl transferase
GGTI: Geranylgeranyl transferase inhibitor
HBSS: Hank’s balanced salt solution
HMG-CoA: 3-Hydroxy-3-methyl-glutaryl-coenzyme A
MBP: Myelin basic protein
MVA: Mevalonic acid
NMO: Neuromyelitis optica
PFA: Paraformaldehyde
PI-PLC: Phosphatidylinositol-specific phospholipase C
PKC: Protein kinase C
